# Phrenic Nerve Interventions for the Prevention and Management of Prolonged Air Leak and Residual Pleural Space After Pulmonary Resection: A Structured Narrative Review

**DOI:** 10.3390/medicina62101863

**Published:** 2026-09-25

**Authors:** László Fehér, Mircea Ioan Șandor, Gabriel Veniamin Cozma, Cristian Marius Daina, Timea Claudia Ghitea, Rareș Cristian Daina, Mădălina Diana Fehér

**Affiliations:** 1 Doctoral School of Biomedical Sciences, Faculty of Medicine and Pharmacy, University of Oradea, 1 December Sq., 410081 Oradea, Romania; feher.laszlo01@yahoo.com (L.F.); diana_daina98@yahoo.com (M.D.F.); 2 Department of Surgical Disciplines, Faculty of Medicine and Pharmacy, University of Oradea, 1st University Street, 410087 Oradea, Romania; drims75@yahoo.com; 3 Discipline of Surgical Semiology 1, Thoracic Surgery, University of Medicine and Pharmacy Victor Babes, 300041 Timisoara, Romania; gabriel.cozma@umft.ro; 4 Department of Psycho-Neurosciences and Recovery, Faculty of Medicine and Pharmacy, University of Oradea, 1 December Sq., 410081 Oradea, Romania; cristi_daina@yahoo.co.uk; 5 Pharmacy Department, Faculty of Medicine and Pharmacy, University of Oradea, 1 December Sq., 410081 Oradea, Romania; 6 Faculty of Medicine and Pharmacy, University of Oradea, 1 December Sq., 410081 Oradea, Romania; rares.daina06@gmail.com

**Keywords:** prolonged air leak, residual pleural space, phrenic nerve, pulmonary resection, diaphragmatic elevation, cryoneuroablation, thoracic surgery

## Abstract

*Background and Objectives:* Prolonged air leak (PAL) and residual pleural space (RPS) remain frequent complications after pulmonary resection. Temporary phrenic nerve modulation has been proposed to reduce pleural cavity volume by elevating the ipsilateral hemidiaphragm. *Materials and Methods:* This structured narrative review searched PubMed/MEDLINE, Scopus, and Google Scholar from inception to 30 June 2026 and screened reference lists. Direct evidence was defined as human post-resection studies reporting PAL, air-leak duration, drainage duration, or RPS; shoulder-pain, interscalene-block, historical, and preclinical studies were retained only as indirect mechanistic, technical, or safety context. Randomized trials and case series were appraised using RoB 2 and the JBI case-series tool, respectively. *Results*: Direct clinical evidence comprises three single-center randomized trials and two small uncontrolled case series. Pharmacological blockade has demonstrated recovery after discontinuation in the available clinical studies, and cryoneuroablation trials reported recovery within 30–60 days to three months. Evidence of reversibility and clinical safety remains insufficient for historical mechanical compression, which has been associated with persistent dysfunction, and for botulinum toxin, which has not been evaluated in humans for this indication. The two cryoneuroablation trials reported discordant primary results; differences in patient risk, surgical era, and outcome measurement are hypotheses rather than established explanations. No head-to-head comparison between phrenic techniques or against established space-reduction methods is available. *Conclusions*: Phrenic nerve modulation is biologically plausible and clinically promising in selected research populations, but the evidence is too limited and heterogeneous to support routine use or comparative recommendations. Future multicenter trials should mandate validated PAL-risk stratification, prespecified respiratory-reserve criteria, standardized PAL/RPS outcomes, and time-to-resolution or digital air-leak measures.

## 1. Introduction

Prolonged air leak (PAL) and residual pleural space (RPS) are among the most frequent complications after pulmonary resection, affecting up to 20% and 40% of patients respectively, and are major drivers of extended chest tube duration, hospital stay, and cost [1,2,3]. Established preventive measures—pleural tenting, pneumoperitoneum, and sealants—each carry technical limitations or are not readily reversible [4,5]. An alternative strategy targets the underlying volume mismatch by inducing temporary ipsilateral diaphragmatic dysfunction via the phrenic nerve, elevating the hemidiaphragm to reduce pleural cavity volume and promote pleural apposition [6]. Early phrenic nerve crush successfully reduced pleural space but carried a substantial risk of permanent diaphragmatic paralysis [7]. Although this concept originated from mid-20th-century collapse therapy [8], it subsequently evolved toward reversible techniques, including cryoneuroablation [9], continuous perineural local anesthetic infusion [10,11], and intraoperative phrenic nerve infiltration [12]. Botulinum toxin has also been proposed as a potentially reversible alternative; however, unlike nerve-targeted interventions, it acts at the neuromuscular junction of the diaphragm, evidence is limited to a preclinical study, and reversibility and safety remain unconfirmed in humans for this indication [13]. Although several studies have investigated individual phrenic nerve techniques, the available evidence remains fragmented across small single-center trials, case series, and historical reports. Previous reviews have addressed prolonged air-leak management in general [14], but none has comprehensively compared the full spectrum of phrenic nerve-targeted interventions or critically examined their mechanistic rationale, clinical efficacy, safety profile, and potential role within contemporary thoracic surgery.

To address this fragmentation without implying a mature or comparative evidence base, the present review uses a structured narrative approach and separates three evidence layers: (1) direct human evidence addressing PAL/RPS after pulmonary resection; (2) historical or preclinical evidence relevant to techniques not yet supported by contemporary human data; and (3) indirect evidence from postoperative shoulder-pain and regional-anesthesia studies used only to inform technique, pharmacology, reversibility, and physiological risk. The objectives are to characterize the reported interventions; summarize PAL/RPS, drainage, pain, pulmonary-function, and safety outcomes; appraise the methodological limitations of the direct studies; and define testable priorities for future trials. No indirect study is used to estimate efficacy for PAL/RPS, and no comparative clinical ranking is inferred in the absence of head-to-head trials.

### 1.1. Anatomy of the Phrenic Nerve

A detailed understanding of phrenic nerve anatomy is essential because the effectiveness and safety of all reviewed interventions depend on the anatomical site of nerve modulation. The phrenic nerve arises predominantly from the C3–C5 cervical roots, with C4 providing the principal contribution, and descends along the anterior surface of the anterior scalene muscle before entering the thorax through the thoracic inlet [15,16].

Within the thorax, the phrenic nerve courses anterior to the pulmonary hilum, running between the mediastinal pleura and the fibrous pericardium in close association with the pericardiacophrenic vessels before reaching the diaphragm [15,17]. In addition to its motor function, it provides sensory innervation to the central diaphragm, mediastinal pleura, and pericardium, explaining its role in referred ipsilateral shoulder pain following thoracic surgery [12,15].

Two anatomical characteristics have direct procedural relevance. First, the intrathoracic segment of the nerve is surrounded by a variable layer of pericardial fat, which serves as the target for intraoperative local anesthetic infiltration and perineural catheter placement, allowing effective drug diffusion while minimizing direct nerve trauma [6,11,12]. Second, an accessory phrenic nerve is present in approximately one-third of individuals [18,19]. When this accessory branch joins the main trunk distal to the site of cervical blockade, diaphragmatic paralysis may be incomplete or shorter than expected, particularly with percutaneous cervical approaches [18,19].

### 1.2. Anatomical Site of Intervention

Although phrenic nerve interventions are commonly classified according to their mechanism of action (pharmacological, thermal, or mechanical), they are equally distinguished by the anatomical level at which they are performed. This anatomical distinction largely determines procedural access, feasibility, safety considerations, and clinical application (Figure 1).

Two principal sites of intervention can be identified.

The cervical approach targets the phrenic nerve percutaneously at the level of the anterior scalene muscle under ultrasound guidance, typically around the C6 vertebral level [6,15]. Because this technique does not require thoracic re-entry, it can be performed postoperatively as a bedside rescue procedure, as demonstrated by Patella et al. [6]. However, its proximity to the brachial plexus and carotid sheath increases the risk of transient local anesthetic spread to adjacent neural structures, while the presence of an accessory phrenic nerve may reduce the completeness of diaphragmatic blockade [15,18]. Interestingly, this is the same anatomical level at which transient hemidiaphragmatic paralysis is routinely observed following interscalene brachial plexus block, illustrating the predictable physiological effect intentionally exploited in this setting [20].

The intrathoracic periphrenic approach targets the nerve where it courses over the pericardium within the periphrenic fat pad, anterior to the pulmonary hilum. This common anatomical target underlies the majority of currently available interventions, including single-shot local anesthetic infiltration [12], continuous perineural catheter infusion [10,11], cryoneuroablation [9,21], and mechanical crush or compression [7]. Because these techniques require surgical exposure of the nerve, they are performed intraoperatively and are therefore primarily used for prophylactic rather than rescue indications. Despite employing different mechanisms of action, all act at the same anatomical location, explaining the remarkably consistent diaphragm-elevation effect observed across studies, with the principal differences relating to the duration and reversibility of diaphragmatic dysfunction.

This anatomical classification also helps explain why direct comparisons between techniques remain scarce. Cervical ultrasound-guided blockade and intrathoracic interventions address different stages of patient management—postoperative rescue versus intraoperative prevention—and should therefore be regarded as complementary rather than competing therapeutic strategies.

### 1.3. Pathophysiological Rationale for Phrenic Nerve Modulation

The rationale for phrenic nerve-targeted intervention is based on a sequence of mechanical events linking pulmonary resection to the development and persistence of prolonged air leak (PAL) and residual pleural space (RPS). Following any anatomical lung resection short of pneumonectomy, the remaining lung is often unable to fully occupy the ipsilateral hemithorax, resulting in a residual pleural space whose evolution depends on the balance between pleural fluid clearance and the continued accumulation of air or fluid [2,22]. Radiographic studies indicate that residual pleural space may occur in up to two-thirds of patients after uniportal VATS lobectomy, although it resolves spontaneously in most cases through compensatory mechanisms, including expansion of the remaining lung, mediastinal shift, narrowing of the intercostal spaces, and elevation of the ipsilateral hemidiaphragm [23,24]. Phrenic nerve modulation deliberately accelerates this final physiological mechanism.

The relationship between residual pleural space and persistent air leak is primarily mechanical. Most postoperative air leaks originate from alveolar–pleural fistulas, where communication between the distal lung parenchyma and the pleural cavity persists until adequate pleural apposition is restored [25]. A larger residual pleural space results in reduced contact between the visceral and parietal pleura, delaying spontaneous closure of the fistula. Consequently, phrenic nerve interventions do not directly treat the air leak itself but instead promote its resolution by reducing pleural cavity volume and improving pleural apposition.

This effect is achieved through controlled elevation of the ipsilateral hemidiaphragm. Under normal conditions, diaphragmatic contraction lowers intrathoracic pressure and facilitates lung expansion during inspiration. Temporary interruption of phrenic nerve conduction abolishes active diaphragmatic contraction, allowing passive cephalad displacement of the hemidiaphragm under abdominal pressure [26,27]. The resulting reduction in pleural cavity volume has been consistently demonstrated by fluoroscopic and radiographic imaging across all major phrenic nerve intervention studies included in this review [9,11,12].

Importantly, the physiological consequences of diaphragm elevation can also be quantified objectively. Digital chest drainage systems continuously measure postoperative air-leak flow and intrapleural pressure, and early changes in these parameters have been shown to predict the subsequent duration of postoperative air leak [28]. These observations provide a mechanistic bridge between phrenic nerve modulation, reduction in pleural cavity volume, and the clinically relevant outcomes evaluated throughout this review, namely accelerated air-leak resolution and obliteration of residual pleural space.

## 2. Materials and Methods

### 2.1. Review Design and Evidence Framework

This review was designed as a structured narrative synthesis rather than a systematic review or meta-analysis. The review question concerned phrenic nerve or diaphragmatic interventions used to prevent or manage PAL and/or RPS after pulmonary resection. Evidence was prespecified in three layers: direct PAL/RPS clinical evidence; historical or preclinical technique evidence; and indirect mechanistic, pharmacological, or physiological evidence. Only the first layer was used to discuss clinical efficacy.

### 2.2. Information Sources and Search Strategy

PubMed/MEDLINE, Scopus, and Google Scholar were searched from database inception to 30 June 2026, with backward reference screening of included studies and relevant reviews. The PubMed strategy was: ((“phrenic nerve”[Title/Abstract] OR diaphragm*[Title/Abstract]) AND (“prolonged air leak”[Title/Abstract] OR “residual pleural space”[Title/Abstract] OR “post-lobectomy space”[Title/Abstract] OR “pleural space”[Title/Abstract]) AND (“pulmonary resection”[Title/Abstract] OR lobectom*[Title/Abstract] OR segmentectom*[Title/Abstract] OR “thoracic surgery”[Title/Abstract])) OR ((“phrenic nerve”[Title/Abstract]) AND (block*[Title/Abstract] OR infiltrat*[Title/Abstract] OR catheter*[Title/Abstract] OR cryoneuroablation[Title/Abstract] OR cryoneurolysis[Title/Abstract] OR cryotherap*[Title/Abstract] OR crush*[Title/Abstract] OR compress*[Title/Abstract] OR “botulinum toxin”[Title/Abstract])). A separate contextual string combined “phrenic nerve” with “shoulder pain”, interscalene, paresis, paralysis, pharmacology, or pulmonary function. Equivalent title/abstract/keyword syntax was used in Scopus. In Google Scholar, the same phrase combinations were screened in relevance order until successive result pages yielded no new eligible technique or evidence category.

### 2.3. Eligibility Criteria and Study Selection

Direct clinical studies were eligible when they enrolled patients undergoing pulmonary resection, intentionally modulated the phrenic nerve or diaphragm, and reported PAL incidence or duration, air-leak resolution, chest-tube duration, or RPS. Randomized trials, non-randomized comparative studies, case series, and case reports were eligible; the single-patient report was retained only as a technical antecedent and was not included in the main evidence table. Historical mechanical reports and animal botulinum toxin studies were retained in separate evidence layers because contemporary human evidence was absent. Indirect studies were eligible only when they informed intervention technique, dose, reversibility, adverse effects, or respiratory physiology; they were excluded from all PAL/RPS efficacy inferences. Duplicate reports, studies without extractable intervention-specific information, and publications unrelated to pulmonary resection or the prespecified contextual questions were excluded. Non-English articles were eligible when relevant data could be translated and extracted.

### 2.4. Data Extraction and Critical Appraisal

For each direct study, the authors extracted design, sample size, surgical population, indication, intervention timing and dose, PAL/RPS and drainage outcomes, respiratory outcomes, recovery of diaphragmatic function, and adverse events. Randomized trials were assessed with the revised Cochrane Risk of Bias tool (RoB 2) [29] and case series with the Joanna Briggs Institute case-series checklist [30]. Appraisal focused on randomization and concealment, deviations from intended intervention, missing data, outcome measurement, selective reporting, consecutive inclusion, completeness of reporting, and the absence of a comparator. The resulting judgments are summarized in Table 1. Because of clinical and methodological heterogeneity and the very small number of studies, no meta-analysis or cross-technique effect ranking was attempted.

### 2.5. Transparency of the Selection Process

Figure 2 reports the search concepts, eligibility pathway, and evidence layers. The original targeted search was not prospectively registered or logged with record-level PRISMA counts, and screening was not conducted in duplicate. Therefore, numerical counts of all records retrieved, deduplicated, and excluded could not be reconstructed without creating false precision. This limitation is disclosed rather than retrospectively presenting the review as systematic.

## 3. Synthesis of the Evidence

### 3.1. Overview of the Available Evidence

The direct clinical foundation is small and heterogeneous: three single-center randomized trials and two uncontrolled case series (Table 1). One case report was retained only as a technical antecedent. Cryoneuroablation is the only technique evaluated in more than one randomized trial, and those trials reported discordant primary results. Mechanical compression is supported mainly by historical reports, while botulinum toxin remains preclinical. These evidence layers are not equivalent and are kept separate throughout the synthesis [31,32,33,34,35,36].

### 3.2. Pharmacological Phrenic Nerve Modulation

Pharmacological modulation comprises single-shot intraoperative infiltration and continuous perineural infusion. Direct PAL/RPS evidence is limited to one small randomized trial and two uncontrolled series; these studies support technical feasibility and documented recovery after discontinuation but do not establish comparative effectiveness or safety in broader post-resection populations.

In the high-risk single-center trial by Trabalza Marinucci et al. [12], intraoperative ropivacaine infiltration was associated with lower PAL incidence, shorter drainage duration, greater early RPS obliteration, and lower pain scores than standard care, while length of stay did not differ. The magnitude of this single-center signal, unequal allocation, absence of a sham procedure, and sensitivity of PAL classification to the drainage protocol require external replication before clinical adoption.

The small series by Carboni et al. [11] established that an intraoperatively placed catheter could produce controllable paralysis with recovery after infusion stopped, but it was not designed to demonstrate efficacy. Patella et al. [6] reported air-leak resolution after postoperative rescue blockade in ten patients with established leak and RPS; without a comparator, the intervention effect cannot be separated from spontaneous resolution or co-interventions. Clavero et al. [10] remains a technical antecedent rather than efficacy evidence.

### 3.3. Local Anesthetic Agents and Future Pharmacological Strategies

Ropivacaine, lidocaine, and bupivacaine are the only local anesthetics reported in the direct or technical-antecedent PAL/RPS literature (Table 2A). Recovery after infusion discontinuation or follow-up was reported for the pharmacological techniques studied, but the small samples do not define uncommon adverse-event rates or safety in patients with marginal respiratory reserve. Approximate single-injection durations for untested agents are derived from the regional-anesthesia literature and are explicitly labeled as extrapolated.

No study has evaluated dexamethasone, dexmedetomidine, epinephrine, liposomal bupivacaine, levobupivacaine, or mepivacaine for intentional PAL/RPS-directed phrenic blockade. Their proposed roles in Table 2 are hypothesis-generating only and should not be interpreted as dosing recommendations.

Studies of phrenic blockade for postoperative shoulder pain and interscalene regional anesthesia provide indirect information on technical feasibility, dose-dependent paresis, recovery, and respiratory physiology [37,38,39,40,41,42,43,44,45,46,47,48,49,50,51,52,53,54,55,56,57,58,59,60,61,62]. They do not test PAL/RPS efficacy and cannot establish the safety of intentionally reducing diaphragmatic function after lung resection, particularly in patients with limited postoperative pulmonary reserve [63,64,65,66,67,68,69,70,71,72,73].

Table 2 therefore separates reported PAL/RPS applications from indirect or extrapolated pharmacological information. Extrapolated entries are retained to define research questions, not to support current clinical use.

### 3.4. Cryoneuroablation

Cryoneuroablation produces cold-induced axonotmesis with delayed recovery and is the only technique evaluated in two randomized PAL/RPS trials [9,21]. The trials are summarized once in Table 1; subsequent discussion focuses on uncertainty rather than repeating their numerical findings.

Pan et al. [9] reported lower PAL and RPS incidence and shorter drainage duration, whereas Meng et al. [21] found no statistically significant difference in its primary early-leak or PAL endpoints. A secondary interval-based analysis in the latter trial suggested faster leak resolution in selected participants. Recovery of diaphragmatic position was reported within 30–60 days in one trial and near baseline by three months in the other.

With only two single-center trials, no definitive explanation for the discordance is possible. Differences in baseline PAL risk, open versus contemporary VATS practice, inclusion of bilobectomy, sample size, and outcome measurement are plausible hypotheses that should be tested prospectively; they should not be interpreted as proof of methodological rather than biological inconsistency.

### 3.5. Mechanical and Emerging Techniques

Mechanical crush or compression has mainly historical support and cannot be assumed to be reversible. Persistent diaphragmatic dysfunction, including permanent paralysis, has been reported [7]. In the absence of contemporary comparative studies and a controllable duration of effect, this approach should not be presented as a routine adjunct.

Botulinum toxin acts at the diaphragmatic neuromuscular junction rather than directly on the phrenic nerve. Evidence is limited to one rabbit study reporting diaphragmatic elevation [13]. Human dosing, duration, reversibility, respiratory consequences, safety, and PAL/RPS efficacy are all unconfirmed; the technique remains preclinical.

### 3.6. Digital Drainage Systems and Assessment of Air-Leak Resolution

The method used to measure postoperative air leak has important implications for interpreting the effectiveness of phrenic nerve interventions. Conventional water-seal drainage systems rely on visual assessment of bubbling, an inherently subjective approach that is prone to interobserver variability and unable to distinguish between a persistent high-flow leak and one that is gradually resolving [63,64]. In contrast, digital drainage systems provide continuous quantitative measurements of air-leak flow, allowing objective assessment of both leak magnitude and its temporal evolution [64,65].

Although randomized studies comparing digital and conventional drainage systems have reported only modest differences in chest tube duration or hospital stay [66,67,68], the principal advantage of digital systems lies in their ability to accurately characterize air-leak dynamics. Continuous measurements obtained during the early postoperative period can predict prolonged air leak more reliably than conventional binary endpoints based on the presence or absence of leakage after a predefined number of postoperative days [27,69,70]. Consequently, digital drainage provides insight into the rate of leak resolution rather than simply documenting whether prolonged air leak has occurred.

The cryoneuroablation trials illustrate why outcome definition matters but do not establish that interval-based findings reconcile their primary results. Pan et al. [9] reported lower binary PAL incidence, whereas Meng et al. [21] did not; the latter study’s secondary interval analysis generated the hypothesis that an intervention might alter the trajectory of leak resolution without changing a binary threshold outcome.

For future clinical studies, routine incorporation of digital drainage systems may therefore represent more than a methodological refinement. By capturing continuous air-leak trajectories instead of dichotomous outcomes, digital monitoring could provide a more sensitive assessment of treatment response and facilitate more accurate comparisons between different phrenic nerve-targeted interventions.

### 3.7. Respiratory and Functional Consequences of Temporary Hemidiaphragmatic Paralysis

The intended physiological effect of these interventions is unilateral diaphragmatic dysfunction, but the duration and reversibility differ by technique. Temporary loss of diaphragmatic contraction can reduce vital capacity, FEV_1_, and functional residual capacity and may worsen basal atelectasis or ventilation–perfusion mismatch [26,27]. Recovery has been documented in the small pharmacological studies and in both cryoneuroablation trials, whereas mechanical injury may persist and botulinum toxin recovery in this indication is unconfirmed.

Most quantitative information on respiratory effects derives from interscalene-block and regional-anesthesia studies [20,71,72,73]. These data support biological plausibility and dose–response concepts but are indirect: they involve different procedures, indications, monitoring, and baseline pulmonary risk and therefore cannot establish post-resection safety.

The clinical paradox is central. Patients with emphysema, incomplete fissures, extensive resection, or low predicted postoperative function may have the highest PAL risk yet the least reserve to tolerate hemidiaphragmatic paralysis. The small direct studies do not define a safe lower threshold for FEV_1_, diffusing capacity, predicted postoperative function, oxygenation, or contralateral diaphragm performance. Consequently, a favorable short-term safety signal in selected study participants should not be generalized to marginal candidates [74,75,76].

Until prospective thresholds are established, reversibility and titratability should be treated as research-design advantages rather than proof of clinical superiority. Future trials should prespecify respiratory-reserve exclusions and stopping criteria; measure baseline and serial FEV_1_, diffusing capacity, oxygenation, dyspnea, and diaphragm excursion; and report respiratory adverse events by validated PAL-risk stratum.

The mechanistic rationale, separated evidence layers, and future research priorities are summarized in Figure 3.

## 4. Discussion

### 4.1. Evidence Hierarchy: Direct and Indirect Evidence

The direct PAL/RPS evidence consists of three single-center randomized trials and two small uncontrolled series. These studies differ in indication (prevention versus rescue), surgical era, baseline risk, intervention duration, and outcome definition. Historical mechanical reports and preclinical botulinum toxin data answer different questions, while shoulder-pain and interscalene-block studies inform only mechanism, dosing, recovery, and physiological risk. Keeping these layers separate prevents indirect evidence from inflating the apparent clinical maturity of PAL/RPS-directed interventions.

### 4.2. Interpretation of the Current Evidence

Phrenic or diaphragmatic modulation is biologically plausible because hemidiaphragmatic elevation can reduce pleural cavity volume and promote pleural apposition. Biological plausibility, however, is not equivalent to demonstrated clinical benefit. Direct evidence is insufficient to determine which patients benefit, whether benefits outweigh respiratory risk, or whether any phrenic technique improves outcomes compared with established space-reduction or leak-management strategies.

No phrenic technique has been compared head to head with another, and none has been directly compared with pleural tenting, pneumoperitoneum, sealants, or endobronchial approaches. Table 3 is therefore an orientation table describing mechanisms, access, and evidence gaps; it is not a comparative-effectiveness matrix and cannot support a treatment hierarchy.

### 4.3. Sources of Clinical Heterogeneity and Uncertainty

The two cryoneuroablation trials reached different primary conclusions. With only two single-center studies, the cause of this discordance cannot be determined. Any explanation based on case mix, surgical era, baseline risk, or outcome measurement remains hypothesis-generating.

Pan et al. [9] included an older open-surgery cohort with bilobectomies, whereas Meng et al. [21] evaluated contemporary VATS lobectomy in an unselected population. These differences could alter baseline PAL risk and detectable effect size, but no patient-level reanalysis or direct comparison demonstrates that they explain the different results. Similarly, the positive trial by Trabalza Marinucci et al. [12] enrolled a validated high-risk population, suggesting—but not proving—that baseline risk may modify treatment effect.

Future trials should test effect modification prospectively by mandating validated PAL-risk scoring before randomization and prespecifying strata for procedure type, emphysema, fissure completeness, predicted postoperative FEV_1_ and diffusing capacity, and intraoperative air leak. Without this design, apparent subgroup benefit will remain inferential.

Outcome heterogeneity is also important. Binary PAL thresholds may miss differences in leak trajectory, whereas secondary interval analyses are vulnerable to multiplicity and selective interpretation. Future protocols should prespecify both a standardized binary PAL definition and time-to-leak-resolution or digital flow trajectories, with transparent handling of chest-tube removal criteria.

### 4.4. Clinical Implications

Clinically, phrenic modulation should currently be regarded as an investigational adjunct aimed at pleural-space mismatch, not as established treatment for the fistula itself. The present evidence does not support routine use, a preferred technique, or substitution for established preventive and rescue measures.

Pharmacological blockade is reversible and, with a catheter, titratable in the limited reports; these characteristics may be attractive when respiratory tolerance is uncertain. Cryoneuroablation provides a longer fixed effect, while mechanical compression carries risk of persistent injury and botulinum toxin lacks human evidence. These are mechanistic and practical distinctions, not comparative clinical recommendations.

Any clinical use outside a trial would require multidisciplinary assessment, explicit consideration of respiratory reserve, monitoring for diaphragmatic and gas-exchange deterioration, and a rescue plan. No evidence-based selection threshold or monitoring protocol is currently validated.

### 4.5. Patient Selection

Perhaps the most important unresolved question concerns identification of patients most likely to benefit from phrenic nerve modulation.

Risk enrichment is biologically and statistically plausible because severe emphysema, incomplete fissures, upper-lobe or bilobar resection, and reduced pulmonary function are associated with PAL. Yet these characteristics may also identify patients least able to tolerate an additional reduction in ventilatory capacity.

Accordingly, future trials should not define “high risk” informally. A validated PAL score should be mandatory and reported by stratum, while respiratory eligibility should be defined separately using predicted postoperative FEV_1_ and diffusing capacity, oxygen requirement, hypercapnia, dyspnea, contralateral diaphragm function, and major neuromuscular disease. Reversible or titratable techniques may be preferable research candidates in marginal participants, but this hypothesis requires testing.

Protocols should include prespecified stopping rules for hypoxemia, hypercapnia, intolerable dyspnea, or excessive loss of diaphragm excursion and should report net clinical benefit rather than air-leak outcomes alone.

### 4.6. Position Within Current Surgical Practice

Phrenic interventions must be evaluated within, not outside, the existing PAL/RPS management pathway.

Pleural tenting, pneumoperitoneum, sealants or buttressing, and endobronchial approaches act through different mechanisms and apply to different anatomical and temporal problems. Theoretical applicability of phrenic modulation to apical or basal space does not establish an advantage over these methods.

Because no direct comparison exists, the appropriate present position is equipoise. Comparative trials should use standardized co-interventions and should evaluate a phrenic strategy against contemporary usual care or an established space-reduction method before any claim of clinical positioning is made.

### 4.7. Future Directions

The present review identifies several priorities for future research.

First, multicenter trials should compare a clearly standardized phrenic intervention with contemporary usual care; head-to-head comparisons between phrenic techniques should follow only after a reproducible benefit signal and acceptable respiratory safety are established.

Second, validated PAL-risk stratification should be mandatory before randomization, with prespecified interaction analyses. Trials should also prespecify respiratory-reserve eligibility and stopping criteria, standardize chest-tube and co-intervention protocols, and report binary PAL, time to leak cessation, digital leak trajectories, RPS, drainage duration, length of stay, pain, serial pulmonary function, dyspnea, quality of life, adverse respiratory events, and cost.

Third, techniques without adequate human evidence require staged development. Botulinum toxin needs dose-finding and safety studies before efficacy testing, and mechanical compression should not be revived without compelling evidence of controllable injury and recovery. Pharmacological dosing and catheter protocols require prospective safety evaluation in patients with reduced reserve.

Figure 4 is a hypothesis-generating research framework, not a validated management algorithm. It identifies possible trial entry points and safety checkpoints and must not be interpreted as a recommendation for routine care.

### 4.8. Limitations

This review and its underlying evidence have important limitations. The review used a structured narrative search but was not prospectively registered, was not designed as an exhaustive systematic review, did not use duplicate screening, and did not retain a complete record-level log from which PRISMA-style retrieval and exclusion counts could be reconstructed. Google Scholar relevance screening is not fully reproducible. Selection and publication bias therefore remain possible despite the explicit search strings and eligibility criteria.

The direct clinical evidence comprises only three single-center randomized trials and two small uncontrolled series. Populations, surgical eras, indications, intervention protocols, drainage systems, PAL definitions, and follow-up differ, and there are no head-to-head or established-treatment comparisons. The positive uncontrolled rescue findings cannot exclude spontaneous resolution, and the randomized trials have design or reporting concerns. Quantitative pooling and comparative ranking would therefore be inappropriate.

Historical mechanical and preclinical botulinum toxin reports were included to map the technique spectrum, not to support clinical efficacy. Shoulder-pain and interscalene-block studies were used only for mechanism, technique, pharmacology, recovery, and respiratory physiology; these indirect data cannot establish PAL/RPS benefit or the safety of intentional hemidiaphragmatic dysfunction after pulmonary resection. Rare harms and outcomes in patients with poor pulmonary reserve remain inadequately characterized.

Accordingly, all clinical implications and the framework in Figure 4 are hypothesis-generating. The review supports a rationale for carefully staged trials, not routine implementation, comparative superiority, or an assumption of reversibility across all techniques.

## 5. Conclusions

Temporary phrenic nerve or diaphragmatic modulation is a biologically plausible approach to reducing pleural-space mismatch after pulmonary resection. Direct clinical evidence, however, is limited to three single-center randomized trials and two small uncontrolled series. Pharmacological blockade and cryoneuroablation have documented recovery in the available studies, whereas mechanical compression may cause persistent dysfunction and botulinum toxin reversibility and safety remain unconfirmed in humans for this indication.

The evidence does not establish a class-wide safety profile, comparative effectiveness, an optimal technique, or superiority to established space-reduction and leak-management strategies. Explanations for discordant cryoneuroablation results based on patient risk, surgical era, or outcome measurement remain hypotheses.

Phrenic modulation should therefore be considered clinically promising but investigational. Before routine implementation, multicenter trials should mandate validated PAL-risk stratification, define respiratory-reserve eligibility and stopping rules, standardize co-interventions and outcomes, and measure both leak resolution and respiratory harm. Until such evidence is available, the proposed framework should be used only to guide research design.

## Figures and Tables

**Figure 1:**
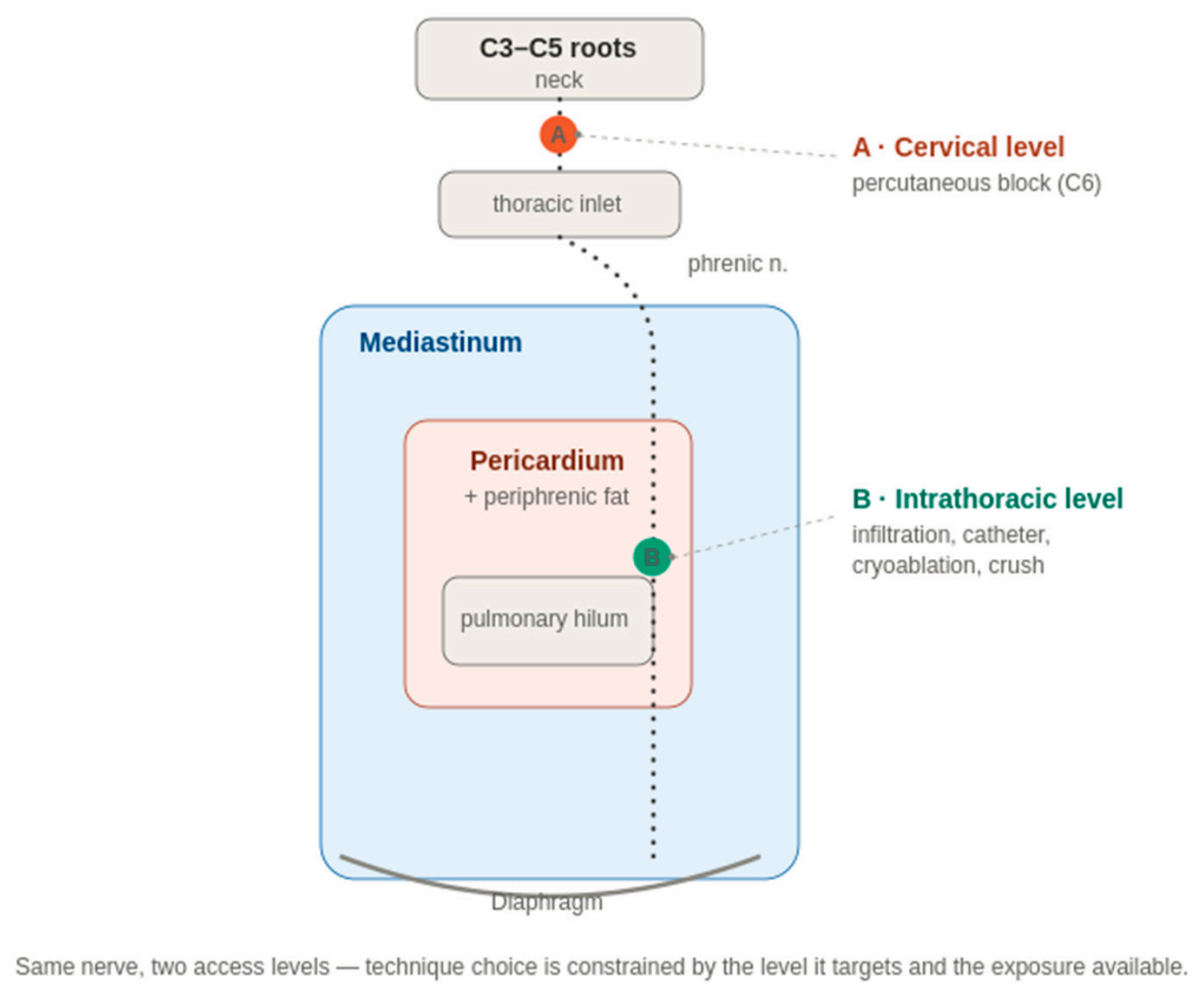
Anatomical targets of phrenic nerve interventions following pulmonary resection. The phrenic nerve originates from the C3–C5 cervical roots, descends through the neck and thoracic inlet, and courses anterior to the pulmonary hilum within the periphrenic fat overlying the pericardium before reaching the diaphragm. Phrenic nerve-targeted interventions are performed at two distinct anatomical levels: (**A**) cervical ultrasound-guided percutaneous blockade, primarily used for postoperative rescue management, and (**B**) the intrathoracic periphrenic approach, which serves as the target for intraoperative local anesthetic infiltration, continuous perineural catheter infusion, cryoneuroablation, and mechanical compression. Botulinum toxin is not illustrated because it acts at the neuromuscular junction of the diaphragm rather than directly on the phrenic nerve.

**Figure 2:**
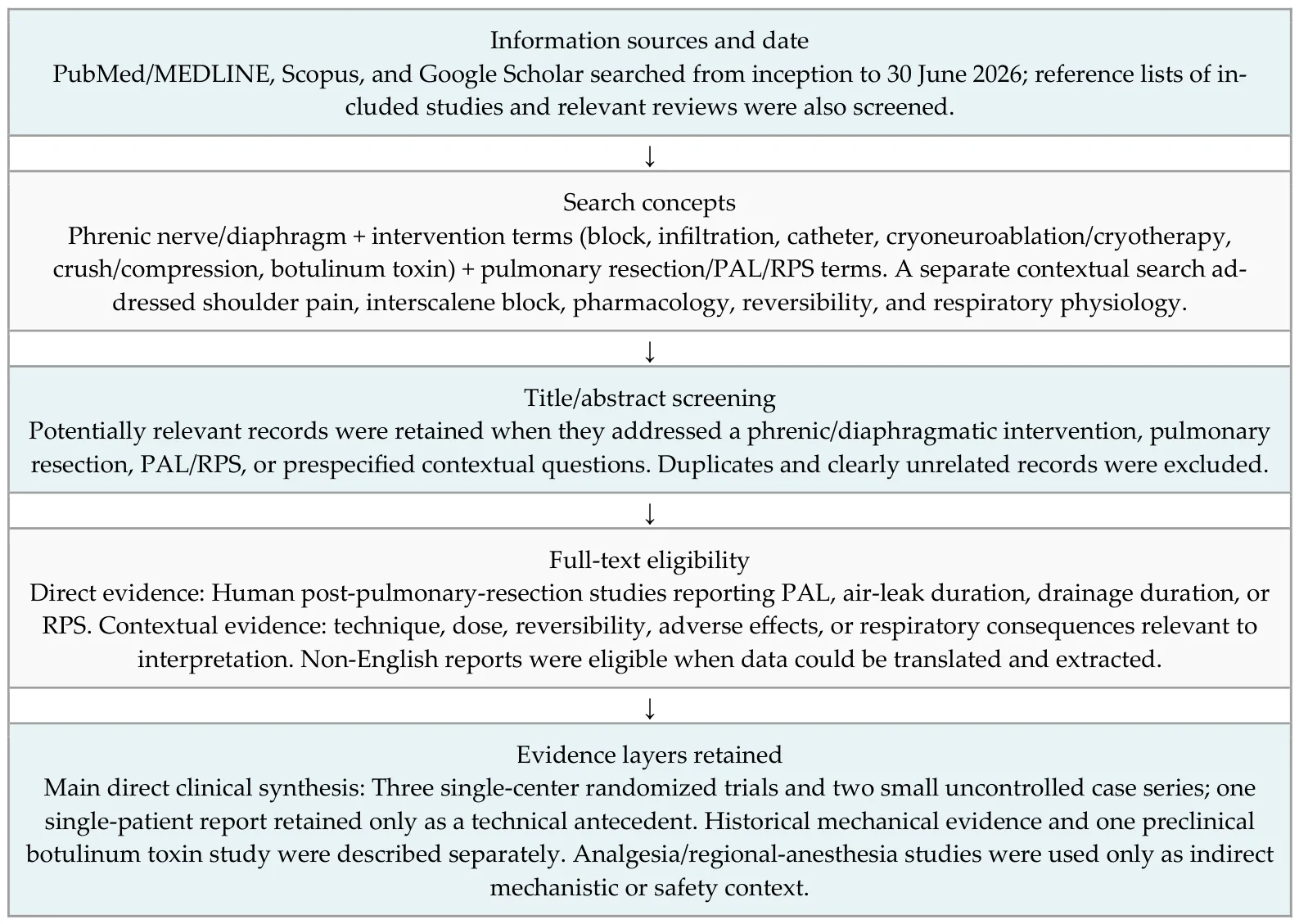
Transparent evidence-identification and selection pathway for this structured narrative review. Because the original targeted search was not prospectively logged as a systematic review, PRISMA-style numerical counts of all retrieved, deduplicated, and excluded records could not be reconstructed reliably; no retrospective counts were invented. This limitation is reported explicitly.

**Figure 3:**
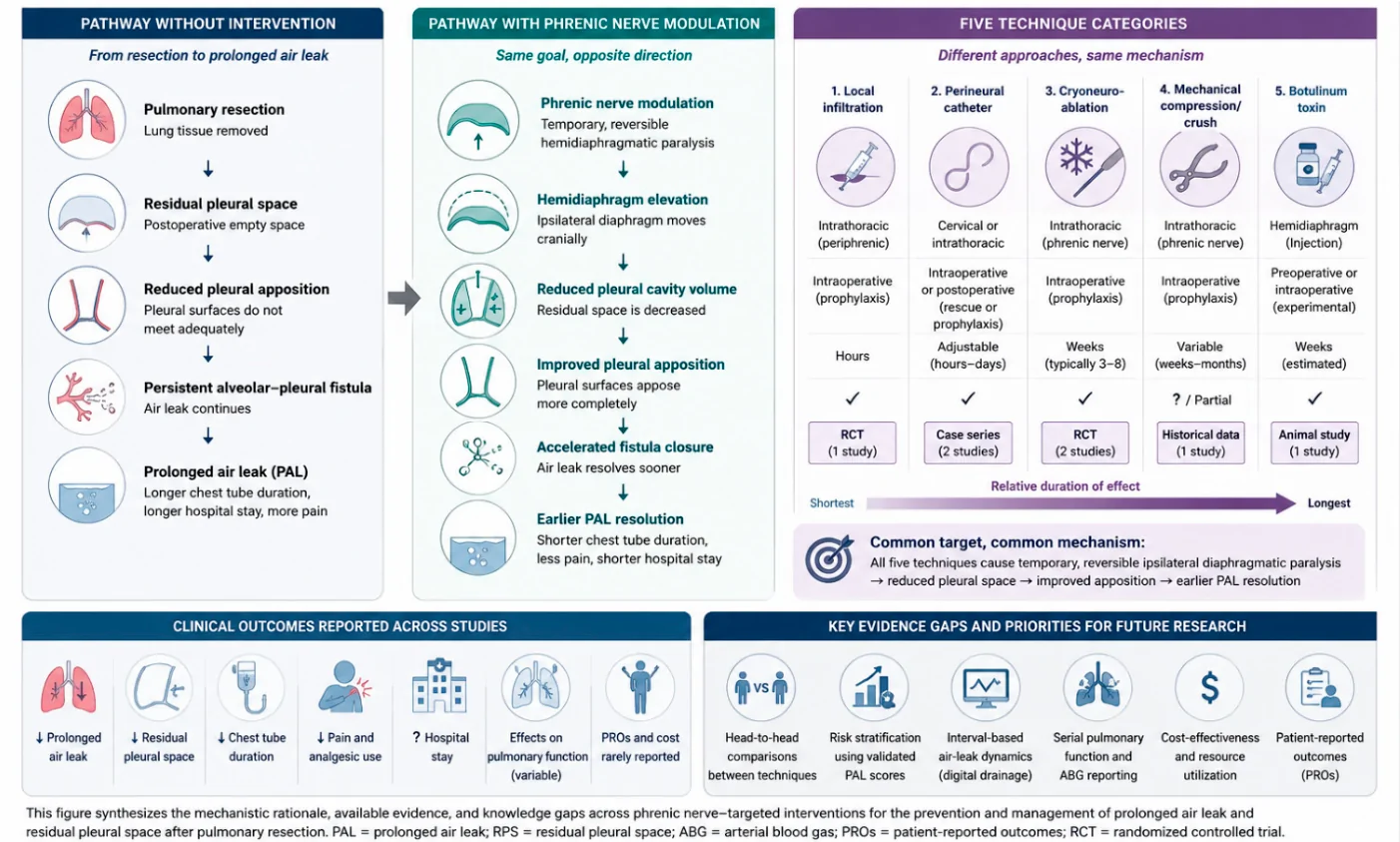
Mechanistic framework, current evidence, and future research priorities for phrenic nerve-targeted interventions in prolonged air leak (PAL) and residual pleural space (RPS). The left panel illustrates the pathophysiological sequence leading from pulmonary resection to prolonged air leak in the absence of intervention. The central panel summarizes the shared mechanism of phrenic nerve modulation, whereby temporary ipsilateral hemidiaphragmatic elevation reduces pleural cavity volume, improves pleural apposition, and accelerates closure of alveolar–pleural fistulas. The right panel maps the five intervention categories according to anatomical target, timing, reported or uncertain reversibility, and level of evidence. The lower panels summarize the reported clinical outcomes and highlight the major knowledge gaps that should be addressed in future multicenter, risk-stratified clinical trials. The figure is explanatory and does not imply comparative effectiveness or readiness for routine use.

**Figure 4:**
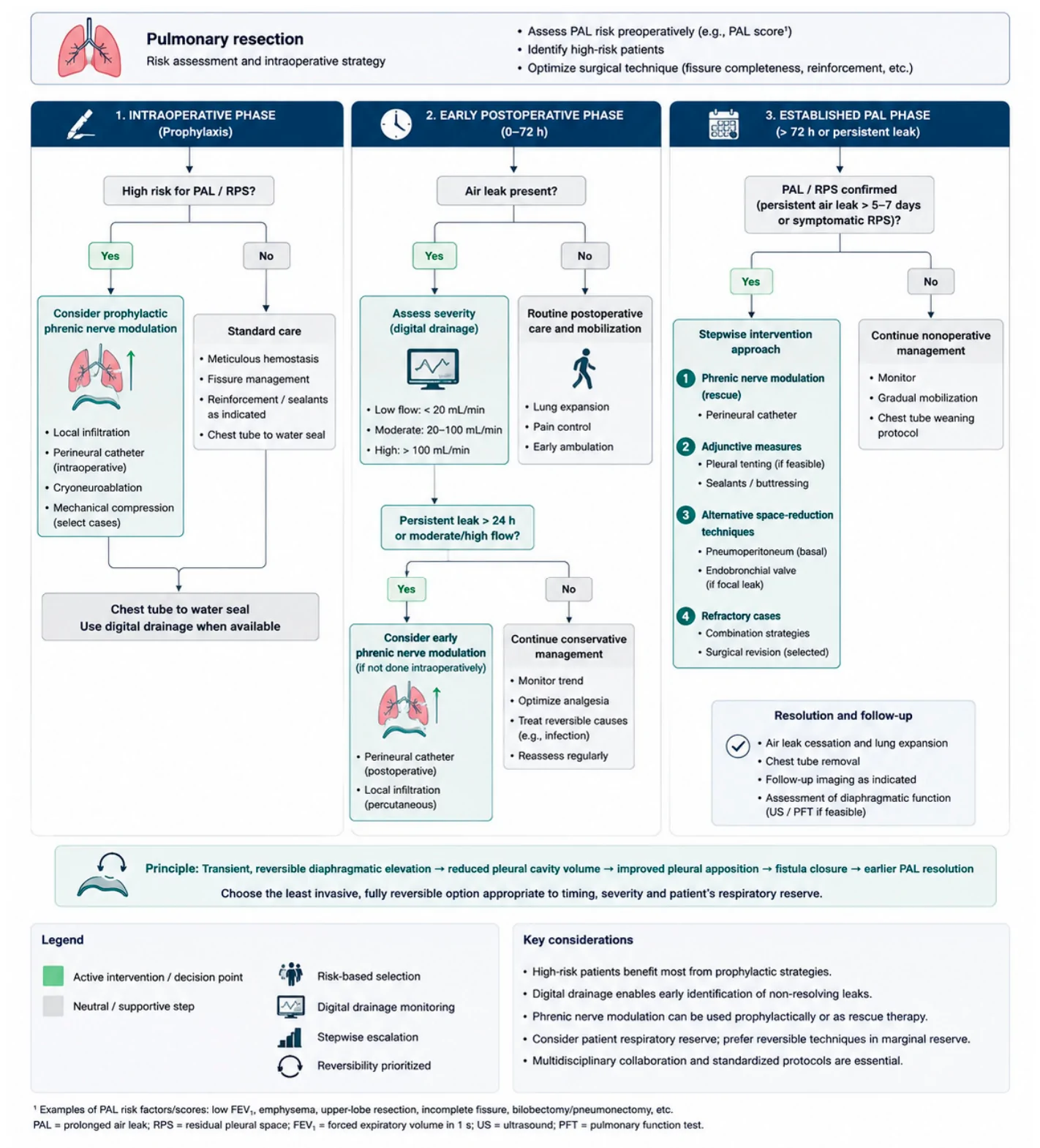
Hypothesis-generating research framework for integrating phrenic nerve-targeted interventions into the prevention and investigation of prolonged air leak (PAL) and residual pleural space (RPS) after pulmonary resection. The framework summarizes the potential role of phrenic nerve modulation across the perioperative course, from intraoperative risk assessment and prophylactic intervention to postoperative monitoring and management of established PAL. Gray boxes indicate standard supportive measures, whereas teal boxes represent potential decision points for phrenic nerve-targeted interventions. The framework is conceptual, derived from the currently available evidence, and is intended to support future clinical investigation rather than provide validated recommendations for routine practice. It is not a validated clinical algorithm and does not recommend routine phrenic intervention.

**Table 1:** Characteristics and outcomes of included evidence.

Study (Year, Country)	Technique	Design; *n*	Population/Indication	Intervention (Timing; Agent/Dose)	Primary Outcomes (PAL/Chest Tube/RPS)	Secondary Outcomes & Safety	Evidence Layer and Structured Appraisal
Trabalza Marinucci 2025 [12], Italy	Single-shot intraoperative infiltration	Prospective RCT (1:2); n = 60 analyzed (A = 22, B = 38)	High-risk PAL (2019 STS criteria); lobectomy/anatomic segmentectomy for malignancy; single center	Intraoperative; ropivacaine 10 mg/mL into perineurotic pericardial fat	PAL 18% vs. 84.2% (*p* < 0.001); chest tube 10.2 vs. 15.8 d (*p* < 0.001); complete RPS elimination POD1 82% vs. 0% (*p* < 0.001)	NRS pain 24 h 3.82 vs. 5.42 (*p* = 0.004), 72 h 3.20 vs. 4.68 (*p* = 0.001); shoulder pain 18% vs. 63% (*p* = 0.001); LOS NS (*p* = 0.080); no procedure-related complications; reversible	Direct pal/rps evidence. RoB 2: some concerns—small single-center trial, unequal allocation, no sham injection/blinding, and outcome sensitivity to digital-drain criteria. Positive efficacy signal requires external replication.
Carboni 2008 [11], Switzerland	Continuous perineural catheter (intraoperative, open placement)	Prospective feasibility/proof-of-principle case series; n = 6	Right-sided lobectomy/bilobectomy; predicted postop FEV_1_ > 1300 mL; 4 NSCLC, 2 pulmonary metastases; single center	Intraoperative; epidural catheter in periphrenic pericardial fat; lidocaine 1% at 3 mL/h (30 mg/h)	Diaphragmatic paralysis confirmed 6/6 by fluoroscopy (POD2); mean drainage 8 d (range 4–18); air leak in 3/6, prolonged (18 d) with persistent RPS in 1	FEV_1_ to ~54% POD1, recovered to ~67% pre-discharge; 2/6 transient hemodynamically irrelevant atrial fibrillation; prompt diaphragm recovery after discontinuation; no allergic/catheter complications	Direct feasibility evidence. JBI appraisal: serious limitations—uncontrolled n = 6 series, selected patients, no efficacy comparator, and feasibility rather than effectiveness objective.
Patella 2018 [6], Switzerland	Continuous perineural catheter (postoperative, percutaneous)	Prospective single-arm case series; n = 10	Established air leak > 200 mL/min at POD3 + radiographic RPS; mixed resections (7 lobectomy, 2 bilobectomy, 1 wedge); single center	Postoperative; US-guided percutaneous phrenic block at C6; ropivacaine 0.2% at 3 mL/h until leak cessation	Air leak resolved 10/10 at mean 3 ± 1.16 d; complete RPS obliteration 8/10 (small asymptomatic space in 2/10)	No peri-/post-procedural complications; full hemidiaphragm recovery after infusion stopped; no surgery-related readmissions at 2 wk	Direct rescue evidence. JBI appraisal: serious limitations—uncontrolled n = 10 series, no counterfactual, mixed resections, and outcomes vulnerable to natural recovery and selection bias.
Pan 2017 [9], China	Cryoneuroablation	Prospective RCT; n = 207 (cryo = 104, control = 103)	Lobectomy/bilobectomy + systematic mediastinal node dissection; single center; 2009–2013	Intraoperative phrenic nerve cryoneuroablation after resection	PAL 1 vs. 9 (*p* < 0.01); RPS 2 vs. 9 (*p* < 0.05); total drainage 1024 vs. 1520 mL (*p* < 0.05); drainage duration 3.2 vs. 4.3 d (*p* < 0.01)	No difference in hospital stay, new drain requirement, or empyema; diaphragmatic paralysis reversed within 30–60 d	Direct pal/rps evidence. RoB 2: some concerns—single center, older surgical era, limited reporting of allocation concealment/blinding, and no contemporary comparator.
Meng 2025 [21], China	Cryoneuroablation (cryotherapy)	RCT (1:1); n = 108 (54/54)	VATS lobectomy + systematic lymphadenectomy for lung cancer; unselected; single center; 2023–2025	Intraoperative phrenic nerve cryotherapy	24 h air leak 29.6% vs. 31.5% (NS); PAL 14.8% vs. 20.4% (NS)	Among intraop-leak patients, faster resolution < 24 h (46.7% vs. 32.0%) and pre-PAL (50.0% vs. 35.3%, *p* < 0.001); diaphragm near-baseline by 3 mo	Direct pal/rps evidence. RoB 2: some concerns—single center, unselected population, limited power for PAL, and secondary/subgroup findings that require confirmation.

Group comparisons are intervention versus control. The table includes the five human clinical studies in the main synthesis. RoB 2 [29] was used for randomized trials and the JBI case-series checklist [30] for uncontrolled series. Appraisal labels indicate limitations of the evidence, not pooled certainty ratings. Clavero et al. [10] was retained as a single-patient technical antecedent but not tabulated. Historical mechanical compression [7] and preclinical botulinum toxin [13] are described separately and are not treated as clinical efficacy evidence. PAL, prolonged air leak; RPS, residual pleural space; RCT, randomized controlled trial; POD, postoperative day; VATS, video-assisted thoracoscopic surgery; US, ultrasound; NRS, numeric rating scale; LOS, length of stay; NS, not significant; STS, Society of Thoracic Surgeons; FEV_1_, forced expiratory volume in 1 s; NSCLC, non-small cell lung cancer.

**Table 2:** (**A**) Local anesthetic agents for temporary phrenic nerve blockade: direct, antecedent, and extrapolated evidence. (**B**) Potential pharmacological adjuncts (all evidence indirect/extrapolated).

(A)					
**Agent**	**Reported Use in Phrenic Nerve Interventions**	**Typical Concentration Reported**	Approximate Duration of Block *	**Main Advantages**	**Current Level of Evidence**
Lidocaine	Continuous intraoperative periphrenic catheter (Carboni et al.)	1%, infusion 3 mL/h	1–2 h	Rapid onset; easily titratable; rapid recovery after infusion discontinuation	Direct pal/rps evidence (small case series)
Ropivacaine	Single-shot intraoperative infiltration (Trabalza Marinucci et al.); postoperative cervical catheter (Patella et al.)	1% (infiltration); 0.2% (continuous infusion)	8–14 h (single injection); titratable with infusion	Lower cardiotoxicity than bupivacaine; favorable sensory–motor differential; best documented agent	Direct pal/rps evidence (one RCT + one case series)
Bupivacaine	Original continuous periphrenic catheter technique (Clavero et al.)	0.25%, infusion 5 mL/h	8–14 h	Long duration; historical proof of concept	Technical antecedent only (single case report)
Levobupivacaine	No published phrenic nerve studies	0.25–0.5% (regional-anesthesia estimate)	8–14 h (not phrenic PAL/RPS-specific)	Similar efficacy with reduced cardiotoxicity	Indirect/extrapolated; no phrenic PAL/RPS study
Mepivacaine	No published phrenic nerve studies	1–1.5% (regional-anesthesia estimate)	2–3 h (not phrenic PAL/RPS-specific)	Predictable intermediate duration; rapid recovery	Indirect/extrapolated; no phrenic PAL/RPS study
Liposomal bupivacaine	Not evaluated in phrenic nerve blockade	13.3 mg/mL (regional-anesthesia estimate)	Up to 72 h (not phrenic PAL/RPS-specific)	Catheter-free prolonged drug release	Indirect/extrapolated; no phrenic PAL/RPS study
**(B)**					
**Adjunct**		**Proposed role**	**Evidence in phrenic block**	**Evidence category**	
Dexamethasone		Prolongs duration of single injection by ~4–8 h	Not studied for PAL/RPS-directed phrenic blockade	Indirect/extrapolated	
Dexmedetomidine		Prolongs peripheral nerve block	Not studied for PAL/RPS-directed phrenic blockade	Indirect/extrapolated	
Epinephrine		Reduces systemic absorption	Not studied for PAL/RPS-directed phrenic blockade	Indirect/extrapolated	

Approximate block durations and proposed adjunct effects are taken from the regional-anesthesia literature unless explicitly identified as direct PAL/RPS evidence. They are estimates, not phrenic-specific measurements or clinical recommendations. Human PAL/RPS evidence currently exists only for lidocaine and ropivacaine; bupivacaine is supported by a single technical report. Every other agent or adjunct in this table is extrapolated. * Durations are approximate and vary with dose, concentration, administration technique, and patient characteristics. Estimates derived from regional anesthesia should not be interpreted as established durations of phrenic nerve blockade for PAL/RPS. For continuous infusions, block duration depends on the infusion regimen; the listed hourly ranges do not represent the total duration of catheter-mediated blockade.

**Table 3:** Descriptive orientation to phrenic techniques and established space-reduction alternatives; no comparative effectiveness is implied.

Technique	Mechanism	Reversibility	Application	Evidence Level	PAL Prevention	PAL Treatment	Ease of Use/Access
Single-shot intraoperative infiltration	Pharmacological phrenic blockade (periphrenic fat)	Recovery reported after local anesthetic effect; uncommon events insufficiently characterized	Prophylactic only	One small single-center RCT [12]	Signal in one RCT; not replicated	Not studied	Easy—single injection at resection, no added equipment
Continuous perineural catheter	Pharmacological blockade, titratable duration	Recovery reported after discontinuation in small series	Both (prophylactic origin; rescue in modern series)	Two small uncontrolled series, n = 6 and n = 10 [6,11]	Not directly tested	Signal in uncontrolled rescue series; efficacy uncertain	Moderate—catheter placement + infusion pump
Cryoneuroablation	Thermal axonotmesis	Recovery reported at 30–60 d to 3 mo in two trials	Prophylactic only	2 RCTs: older positive (n = 207), contemporary null (n = 108) [9,21]	Discordant RCT primary results	Not studied	Moderate—cryoprobe, intraoperative exposure
Mechanical crush/compression	Mechanical axonal disruption	Uncertain; persistent dysfunction reported	Prophylactic only	Historical series only, no contemporary comparator [7]	No contemporary efficacy test	Not studied	Easy mechanically, morbidity-limited
Botulinum toxin neurolysis	Chemical NMJ blockade (distal to nerve)	Unconfirmed in humans for this indication	Prophylactic only, preclinical	Single rabbit study only [13]	Not tested in humans	Not tested in humans	Unknown—no human procedural data
Pleural tenting	Mechanical space reduction (apical)	N/A—structural	Prophylactic, apical/upper-lobe only	RCTs + meta-analysis [77,78,79,80]	Supported for selected surgical contexts; no phrenic comparison	No	Moderate—anatomically limited
Pneumoperitoneum	Diaphragm elevation by insufflation (non-neural)	Partial—not immediately reversible	Both prophylactic and rescue	RCTs + case series [5,81,82]	Reported in separate evidence base; no phrenic comparison	Reported in separate evidence base; no phrenic comparison	Moderate—abdominal access required
Sealants/buttressing	Mechanical staple-line sealing	N/A	Prophylactic only	Inconsistent outside severe emphysema [24]	Variable evidence; no phrenic comparison	No	Easy—applied at stapling
Endobronchial valves	Bronchial occlusion (fistula exclusion)	Reversible (removable)	Rescue/treatment only	Multicenter series + systematic reviews [83,84,85,86,87]	No	Salvage evidence; no phrenic comparison	Moderate—bronchoscopy, collateral-ventilation check

The bottom four rows are non-neural comparators included only to show the existing therapeutic landscape. Cross-row comparisons are descriptive and invalid as estimates of relative benefit or harm because populations, indications, designs, and outcomes differ.

## Data Availability

The original contributions presented in this study are included in this article. Further inquiries can be directed to the first authors.

## Abbreviations

The following abbreviations are used in this manuscript:

Abbreviation	Definition
BTX-A	*Botulinum Toxin* Type A
CI	Confidence Interval
CT	Computed Tomography
FEV_1_	Forced Expiratory Volume in One Second
LOS	Length of Hospital Stay
NS	Not Significant
NSCLC	Non-Small Cell Lung Cancer
OR	Odds Ratio
PAL	Prolonged Air Leak
POD	Postoperative Day
RCT	Randomized Controlled Trial
RPS	Residual Pleural Space
RR	Relative Risk
STS	Society of Thoracic Surgeons
US	Ultrasound
VATS	Video-Assisted Thoracoscopic Surgery
NMJ	Neuromuscular Junction
